# Management Strategies for Congestive Heart Failure in Chronic Kidney Disease: Narrative Review

**DOI:** 10.3390/biomedicines14040841

**Published:** 2026-04-07

**Authors:** Pamela Soto-Santillan, Andres Jacobo-Ruvalcaba, Michael Eduard Wasung-de Lay, Oscar Orihuela-Rodriguez

**Affiliations:** 1 Department of Nephrology, Hospital Central Sur de Alta Especialidad, Petroleos Mexicanos, Mexico City 14140, Mexico; pamss1721@yahoo.com (P.S.-S.); mwasungll@gmail.com (M.E.W.-d.L.); 2 Clinical Department of Cardiology, UMAE Hospital de Especialidades, Centro Médico Nacional Siglo XXI, Instituto Mexicano del Seguro Social, Mexico City 06720, Mexico; electrocardiologia.2016@gmail.com

**Keywords:** heart failure, chronic kidney disease, hydro-saline overload, renal replacement therapy

## Abstract

Heart failure (HF) affects approximately 64 million people globally. HF often coexists with chronic kidney disease. HF may affect the heart during diastolic filling, systolic ejection, or both. Conventionally, HF is categorized by left ventricular ejection fraction (LVEF). One of the leading causes of death in chronic kidney disease (CKD) patients of cardiovascular origin increase hospitalizations and worsen quality of life by causing fluid and electrolyte overload. As kidney function declines, increases risk of development of HF in CKD, with a negative impact and worse prognosis in these patients. This narrative review provides healthcare professionals—including nephrologists, car-diologists, internists, and general practitioners—with evidence-based strategies to iden-tify and manage this complex comorbidity, aiming to reduce hospitalization and mor-tality in CKD patients. By synthesizing recent findings on risk stratification, diagnostic modalities, and individualized treatment—particularly for patients undergoing renal replacement therapy—clinicians can enhance volume management and optimize patient outcomes. Considering the increasing prevalence of chronic kidney disease and associated cardiovascular comorbidities, this review addresses pathogenic mechanisms, diagnostic approaches, pharmacological treatments, and dialytic therapy modifications.

## 1. Introduction

Heart failure (HF) is a clinical syndrome characterized by dyspnea, ankle edema, and fatigue, often accompanied by jugular venous distension, pulmonary crackles, or signs of fluid overload [1,2,3]. HF results from structural or functional cardiac abnormalities that increase intracardiac pressure or reduce cardiac output at rest or exercise [4,5]. This clinical condition affects the heart during diastolic filling, systolic ejection, or both. Heart failure is classified by the left ventricular ejection fraction (LVFE) into the following groups:

1. Reduced Ejection Fraction (HFrEF) is defined by a left ventricular ejection fraction (LVEF) of 40% or less.
2. Mildly reduced or improved Ejection Fraction (HFmrEF or HFimpEF) is characterized by a LVEF between 41% and 49%.
3. Preserved Ejection Fraction (HFpEF) is defined by a LVEF of 50% or greater.

By linking LVEF categories directly with therapeutic options and clinical priorities, this framework can support more precise and effective decision-making at the bedside. [6].

Cardiovascular mortality is significantly elevated in patients with chronic kidney disease (CKD). A cohort study demonstrated higher mortality rates among CKD patients compared to those without CKD [7].

There is a meta-analysis and an ARIC cohort study that demonstrates a high incidence of HF in CKD patients [8,9].

As kidney function declines, the risk of developing HF increases. For example, it is estimated that 44% of the population with CKD receiving dialysis is at high risk for HF. Additionally, some cohorts have reported an incidence of Heart Failure with Reduced Ejection Fraction (HFrEF) as high as 50% [10].

The prognosis for patients with HF and CKD is poor and worsens as renal function deteriorates, resulting in a 32% mortality (odds ratio [OR]: 2.34; 95% confidence interval [95% CI]: 2.20 to 2.50) compared to patients without HF [8].

Patients on dialysis have an eight times higher mortality compared to the control group, with an estimated cardiovascular mortality of 43% [11].

Heart failure in patients with chronic kidney disease increases due to the combination of traditional and non-traditional risk factors, including diabetes mellitus (DM), systemic arterial hypertension (SAH), obesity, chronic inflammation, anemia, chronic kidney disease–mineral bone disorder (CKD-MBD), hyperphosphatemia, and vascular calcifications.

The presence of heart failure with CKD is complex; therefore, for effective management, a collaborative, multidisciplinary approach between nephrologists and cardiologists is necessary. For instance, implementing a joint nephrology–cardiology clinical pathway facilitates coordinated assessment, shared decision-making, and seamless transitions between services, enabling rapid evaluation and integrated management of patients presenting with acute volume overload tailored to their complex needs [12].

## 2. Pathophysiology of Chronic Kidney Disease Associated with Heart Failure

One of the mechanisms involved in the development of HF is left ventricular hypertrophy (LVH), which is the main structural alteration in patients with CKD. Abnormalities in structure and function, as well as myocardial fibrosis, develop in the final stages of CKD; however, in the pre-dialysis stages, they are associated with eccentric LVH [13].

**Figure 1:**
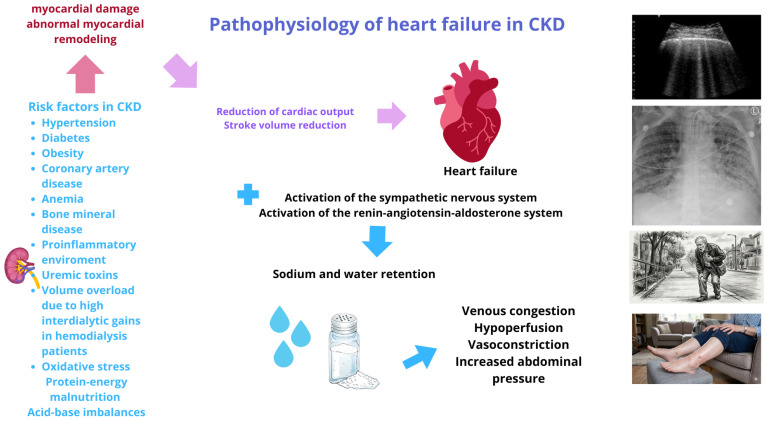
Pathophysiology of heart failure in CKD patients.

The presence of traditional risk factors such as age, smoking, hypertension, dyslipidemia, diabetes, and obesity in patients with CKD increases the risk of heart failure associated with non-traditional factors such as anemia due to reduced erythropoietin production, causing fatigue and impaired oxygen delivery to the myocardium; iron deficiency may exacerbate this condition. Persistent low-grade inflammation prevalent in CKD promotes vascular injury and progressive cardiac dysfunction. Oxidative stress, resulting from increased free radicals and reduced antioxidant defenses, further damages cardiac tissue. The accumulation of uremic toxins, which the kidneys fail to eliminate effectively, exerts direct and indirect cardiovascular toxicity. Disrupted bone and mineral metabolism, including hyperphosphatemia, contributes to vascular calcification and arterial stiffness. Elevated homocysteine levels (hyperhomocysteinemia) and increased albuminuria further predispose CKD patients to cardiac complications. Collectively, these nontraditional mechanisms elucidate the heightened cardiovascular risk associated with CKD [14]. Figure 1 show essential mechanisms involved in the appearance of HF in CKD patients.

Nardin conducted a prospective study comparing patients with CKD and those with hypertension, finding a higher prevalence of LVH in 62.8% of patients with CKD compared with 51.9% in the hypertensive group. At the CKD stage, he observed a greater increase in LV diameter and LV wall thickness [15].

Maqbool et al., in a systematic review of left ventricular alterations in patients with CKD (stages 2 to 5), found concentric remodeling in 11.17% and concentric hypertrophy in 47.05%. In stages 4 and 5, he found a higher incidence of eccentric hypertrophy [16].

After 18 months of peritoneal dialysis, the ventricular mass index increases in up to 62% of patients, with 49% developing heart failure. Altered calcium-phosphorus metabolism, seen even before dialysis and intensified in stage 5 CKD, contributes to vascular calcification and ventricular hypertrophy. [17]. Despite therapeutic interventions using calcimimetics and calcium or non-calcium binders, relevant limitations persist, as clinical trials evaluating paricalcitol versus cardiac parameters of ventricular hypertrophy (e.g., OPERA and PRIMO trials) found no significant changes in ventricular geometry [18,19].

The increase in ventricular mass is the main structural abnormality in CKD cardiomyopathy, accompanied by histological changes such as severe intramyocardial arteriolar thickening, reduced capillary density (decreased myocardial capillary perfusion), hypertrophic and bizarre myocytes, and myocardial interstitial fibrosis [20,21]. The rise in fibrotic interstitial, characterized by increased type I-III collagen, reduces contractility and alters diastolic function, leading to exercise intolerance. Consequently, heart failure and cardiac arrhythmia may develop [22].

The risk factors associated with left ventricular hypertrophy and myocardial fibrosis in CKD associated with cardiomyopathy are divided into three categories as follows:

Preload-dependent factors (volume overload).

Afterload-dependent factors (pressure overload).

Non-hemodynamic factors associated with CKD (cardiomyopathy).

Preload-related factors include increased intravascular volume, anemia, and greater blood flow through arteriovenous fistulas in hemodialysis patients [23].

In patients with arteriovenous fistulas, cardiac output increases by 20% due to volume overload. This leads to higher left ventricular mass index, larger left ventricular end-diastolic and end-systolic diameters, and increased left atrial volume. Manifestations of heart failure occur. A high-flow arteriovenous fistula is associated with dilated left ventricular dimensions and volumes, as well as impaired left ventricular systolic function [24].

Afterload-dependent factors include increased arterial resistance and reduced arterial flexibility due to vascular calcification, leading to left ventricular thickening [25]. Activation of the renin–angiotensin–aldosterone system occurs in all these processes. However, the angiotensin and aldosterone systems specifically cause thickening and scarring of the heart muscle [26].

One of the non-traditional factors for the development of congestive heart failure is alterations in bone and mineral metabolism, orchestrated by fibroblast Growth Factor 23 (FGF-23), a phosphaturic hormone that plays a relevant role in left ventricular hypertrophy, which, together with the parathyroid hormone (PTH), regulates phosphate recycling and calcitriol synthesis in the active metabolite of vitamin D in the kidney [27]. FGF is a family of peptides secreted by osteoblasts and osteocytes in bone. It acts on the kidney, parathyroid, bone, and heart. This metabolite is produced in the kidney by paracrine function, which facilitates the elimination of phosphate by blocking the synthesis of vitamin D and inhibiting phosphate reabsorption in the proximal nephron.

There are other FGF-23 signaling pathways through FGFR3 binding and activation, independent of αKlotho, in CKD patients with high circulating FGF-23 levels. Directly stimulating receptors on myocytes causes left ventricular hypertrophy and promotes fibrosis, which worsens heart failure and increases mortality [28].

Membrane-bound αKlotho is an essential cofactor of FGF-23, thereby involved in the regulation of phosphate and vitamin D metabolism, with cardioprotective effects through the inhibition of calcium channels. αKlotho is regulated by multiple transmembrane factors, including 1–25 OHD, aerobic exercise, ACE inhibitors, and nuclear factor kappa beta [29]. Silva et al. evaluated αKlotho and FGF23 in patients with DM and CKD in the pre-dialysis stage, showing that low αKlotho and high FGF-23 levels were associated with a higher risk of concentric and eccentric hypertrophy [30].

Elevated FGF-23 helps maintain normal serum phosphate in CKD, but higher FGF-23 levels are dose-dependently associated with increased risks of LVH, HF, and mortality in patients with CKD [31]. The increase in FGF-23 levels in patients with CKD is associated with alterations in ventricular mass, which favor hypertrophy associated with HF [32].

## 3. Diagnosis of Heart Failure in Chronic Kidney Disease

The diagnosis of heart failure requires the presence of signs and symptoms such as dyspnea and fatigue, with evidence of structural and/or functional abnormalities [6]. The same criteria for heart failure are used in patients with and without CKD.

Heart failure classification systems developed by organizations such as the European Society of Cardiology, American College of Cardiology, American Heart Association, and the New York Heart Association (NYHA) each provide distinct frameworks. However, these classifications have limitations in patients with chronic kidney disease undergoing intermittent hemodialysis, as dyspnea may result from volume fluctuations rather than heart failure alone. Consequently, the Acute Dialysis Quality Initiative Workgroup has proposed a functional classification system specifically for heart failure in CKD patients on hemodialysis [33]. This system accounts for the timing of evaluation within the dialysis cycle and categorizes heart failure accordingly in this patient population summarize in Table 1.

## 4. Diagnosis and Biomarkers of Heart Failure in CKD

The most commonly reported is dyspnea. This must be classified as exertional, positional (orthopnea), and acute or chronic. Other commonly reported symptoms of HF include chest pain, anorexia, and exertional fatigue. Anorexia is due to hepatic congestion, bowel edema, and reduced blood flow to the splanchnic circulation. Patients may also experience abdominal discomfort due to hepatic congestion or ascites [34].

### 4.1. Framingham Clinical Diagnostic Criteria for Heart Failure

The commonly used Framingham Diagnostic Criteria for Heart Failure require the presence of two major criteria or one major and two minor criteria to make the diagnosis. This clinical diagnostic tool is highly sensitive for HF diagnosis but has relatively low specificity. To diagnose heart failure, the Framingham criteria use major and minor signs see Table 2.

In cardiology and nephrology, the utilization of biomarkers has become essential for diagnosis, risk stratification, and prognostication, facilitating effective communication regarding the patient’s current clinical status [35].

Natriuretic Peptides:B-type Natriuretic Peptide (BNP) and N-terminal proBNP (NT-proBNP) are synthesized from a pre-hormone of 134 amino acids, encoded by the NPPB gene, and they are biologically active molecules. BNP is produced primarily by ventricular cardiomyocytes in response to volume or pressure overload. Circulating BNP and NT-proBNP levels are low in physiologic conditions but increase in HF patients. This biomarker increases arterial vasodilation and natriuresis, exerts anti-hypertrophic and anti-fibrotic effects, and promotes activation of the renin–angiotensin–aldosterone system (RAAS), sympathetic nervous system (SNS), and the endothelin systems [36].

The inactive fragment of B-type natriuretic peptide (Pro-BNP) is produced in response to increased end-diastolic pressure of the atrium or end-diastolic distention of the ventricle. High levels are a predictor of mortality in patients with CKD. In a meta-analysis that includes patients with CKD with HF, the increased levels ofBNP/Pro-BNP were associated with all causes of mortality, cardiovascular mortality, and cardiovascular events [37]. In a multicenter prospective study in patients with CKD plasma pro-BNP and extracellular fluid volume/total body water ratio were predictors of both all-cause and cardiovascular mortality, independently of dialysis modality clinical and biochemical risk factors [38].

NT-proBNP level in participants of four pivotal trials was included with different levels of kidney function; the authors used Poisson regression, and they compared the equivalent NT-proBNP concentration corresponding to an unadjusted event rate of 5 and 10 per 100 patient-years in patients with different levels of GFR. Each doubling in NT-proBNP was associated with a 37% relative increase in the primary outcome:hospitalization for heart failure or cardiovascular death (HR: 1.37; 95% CI: 1.34–1.41) [39].

Standardization and harmonization of biomarker reference values remain insufficient in the chronic kidney disease (CKD) population, necessitating further evaluation across diverse ethnic groups. Various studies have assessed biomarkers such as B-type natriuretic peptide (BNP) in CKD. For instance, Masson et al. reported BNP levels of 769 pg/mL in pre-dialysis patients, compared with levels exceeding 2023 pg/mL in patients with an estimated glomerular filtration rate (eGFR) below 60 mL/min [40]. McCullough et al. documented BNP levels above 200 pg/mL in patients with CKD stage 3 [41]. Horii et al. analyzed the prognostic value of BNP, reporting 90.8 pg/mL in patients with eGFR greater than 30 mL/min versus 157 pg/mL in those with eGFR below 30 mL/min, demonstrating prognostic significance for mortality and major adverse cardiovascular events (MACE) [42]. Fujii et al. compared plasma BNP and NT-proBNP levels and their ratio, revealing that the association between CKD stage and these biomarker levels diminishes with declining renal function (see Table 3).

### 4.2. Troponins

Cardiac troponins (cTnT), including highly sensitive troponin (hs-TnT), are used as prognostic markers of mortality in CKD. The CRIC study shows that cTnT, with high sensitivity, detected in 84% of patients with GFR < 30 mL/min, had 3-fold higher expected hs-TnT compared to subjects with eGFR > 60 [44]. There are other studies that consider that high levels of troponin in asymptomatic hemodialysis patients are complex and the presence of this marker is possibly secondary to the presence of stunned myocardium in hemodialysis [45].

Rehm et al. evaluated troponin levels in patients with CKD who had a glomerular filtration rate less than 60 mL/min, with a median and IQR of 4.7 (3–7.3) [46].

Considering that there are myocardial and non-myocardial causes of troponin elevation, such as myocarditis, endocarditis, pericarditis, cardiopulmonary resuscitation, uncontrolled hypertension, pulmonary thromboembolism, sepsis, severe anemia, and rhabdomyolysis, the study found that there are myocardial and non-myocardial causes of troponin elevation [47].

### 4.3. Galectin

It is part of the galactoside-lectin family, synthesized by macrophages, and interacts specifically with cellular matrix proteins, including laminin, syneixin, and integrins. Galectin is considered a predictor of cardiovascular death [48]. The serial determination of galectin-3 levels has prognostic significance in heart failure with HFr or HFpEF [49]. There is an association between circulating galectin-3 and the risk of heart failure [50]. High galectin-3 levels (≥34.3 ng/mL) were associated with higher incidence rates of cardiovascular events and mortality in patients with CKD and HF with reduced ejection fraction in patients with CKD in hemodialysis [51].

### 4.4. ST2

Suppression of tumorigenicity 2 protein (ST2), a member of the interleukin-1 receptor family, is expressed in various cell types under conditions such as stress and inflammation and plays a significant role in heart failure [52]. Lower serum ST2 levels in patients with heart failure with preserved ejection fraction (HFpEF) compared to those with reduced ejection fraction (HFrEF) may indicate a lesser degree of fibrosis; however, its closer association with clinical outcomes suggests that progressive fibrosis is more prognostically relevant [53]. In patients with chronic kidney disease (CKD), ST2 levels are an independent prognostic factor with superior predictive ability compared with B-type natriuretic peptide (BNP) for all-cause mortality and cerebrocardiovascular events [54]. The prognostic value of soluble ST2 (sST2) is additive to N-terminal pro-B-type natriuretic peptide (NT-proBNP) and high-sensitivity cardiac troponin T (hs-cTnT), with combined use enhancing risk stratification for cardiovascular mortality in the hemodialysis population [55].

### 4.5. GDF-15

GDF-15, also termed macrophage inhibitory cytokine 1 (MIC-1), belongs to the transforming growth factor-β (TGF-β) superfamily proteins, relevant in regulating development, differentiation, and tissue repair in various organs [56,57].

In clinical predictor models, they include levels of BNP, GDF-15, sex, systolic blood pressure, sodium, total cholesterol, and ACEi/ARB treatment as significant variables associated with ventricular assist device implantation and death [58].

### 4.6. CA 125

It is a sensitive but not specific biomarker in patients with CKD and fluid overload; an increase in it has been observed in patients with HFrEF compared with those with preserved ejection fraction and HF [58,59] (Table 4).

### 4.7. Imaging

The echocardiogram plays an important role in the diagnosis and follow-up of HF patients [69]. Cardiac magnetic resonance (CMR) provides a better assessment than echocardiography, which is considered the gold standard evaluation. However, the use of gadolinium brings with it major adverse effects for patients, specifically in those with CKD, for the risk of nephrogenic systemic fibrosis, which limits its use in clinical practice in this susceptible population [70].

## 5. Heart Failure Treatment

Management of heart failure (HF) involves salt and water restriction, blood pressure control, and pharmacological therapies including beta-blockers, mineralocorticoid receptor antagonists, angiotensin-converting enzyme (ACE) inhibitors, angiotensin receptor blockers (ARBs), sodium-glucose cotransporter-2 (SGLT2) inhibitors, and diuretics [71]. Additionally, agents targeting cardiac remodeling, such as angiotensin receptor-neprilysin inhibitors (ARNIs) [72,73] and SGLT2 inhibitors [74,75], are central to treating HF with reduced ejection fraction. The pathophysiology of HF includes maladaptive activation of the renin–angiotensin–aldosterone system (RAAS), counterbalanced by activation of the natriuretic peptide system. Neprilysin inhibition prevents degradation of natriuretic peptides, thereby prolonging their beneficial effects [76,77].

## 6. Treatment of Heart Failure in Chronic Kidney Disease

Despite established pharmacological treatments and the introduction of novel agents, managing heart failure in patients with chronic kidney disease remains a significant clinical challenge.

### 6.1. Beta-Blockers

Multiple studies have shown that beta-blockers (BB) reduce hospitalizations and mortality in patients with HF with preserved and reduced ejection fraction [78,79].

Beta-blockers are a mainstay in the management of reduced and mildly reduced congestive heart failure. Several randomized controlled clinical trials have shown, across different cohorts, that bisoprolol in CIBIS II reduced the risk of all-cause mortality and had a beneficial effect on sudden cardiovascular death [80].

Regarding the use of metoprolol, the MERIT HF trial showed a reduction in the risk of death with an odds ratio (OR) of 0.53–0.81, as well as lower rates of functional class decline [81].

The COPERNICUS trial, which evaluated carvedilol versus placebo, observed a 35% reduction in death and hospitalization in patients with heart failure [82]. The SENIORS study in geriatric patients comparing nebivolol versus placebo in patients with ejection fraction less than 35% showed a protective effect of nebivolol use with HR 0.86 (0.74–0.99) against cardiovascular death, with no effect on overall mortality [83].

The inclusion of patients with CKD is a limitation in relation to controlled clinical trials. Wali et al. evaluated all-cause mortality and cardiovascular mortality in patients with CKD in the pre-dialysis stage. The authors observed reductions in the risk of all-cause mortality, cardiovascular mortality, HF mortality, and HF hospitalization; both patient groups tolerated carvedilol well [84].

In patients with CKD, carvedilol improves ejection fraction in patients with HF, reducing systolic and diastolic volumes compared with placebo [85]. At two years of follow-up, the mortality rate was reduced by up to 49% [86]. A meta-analysis evaluated the effectiveness of BBs in more than 120,000 dialysis patients and demonstrated that they significantly reduce all causes of mortality and hospitalizations [87].

### 6.2. Angiotensin Receptor-Neprilysin Inhibitors (ARNI)

Sacubitril/valsartan (SV) is a neprilysin receptor inhibitor. It forms a complex with sacubitril and valsartan (which blocks angiotensin receptors) in a 1:1 ratio. After administration, it is hydrolyzed by a carboxylesterase to sacubitril. The molecule, along with valsartan, binds to plasma proteins at 94–97% [88,89]. SV inhibits peptide degradation and affects the renin-angiotensin system. Also, the biological effects on the natriuretic peptide system include natriuresis and diuresis, as well as vasodilation and fluid mobilization [90]. In an observational study of 110 patients with HF and hemodialysis who took sacubitril/valsartan, an improvement in EF of 35.1% was observed, and at 12 months of follow-up, it increased to 49.8%, with a reduction in left ventricular mass index from 167.8 g/m^2^. versus 154.4 g/m^2^ and left ventricular end-diastolic diameters from 52.2 mm to 51.4 mm at 12 months, as well as the left ventricular end-systolic diameter from 35.9 mm to 36.9 mm at 12 months [91].

The pharmacology and pharmacokinetics of sacubitril/valsartan were analyzed in patients on peritoneal dialysis (PD), with residual renal function contributing to a lesser extent to the elimination of the active substance. The dose of 100 mg of sacubitril/valsartan is safe and effective in patients with PD with complications of arterial hypertension and heart failure [92,93].

The active metabolite LBQ657 and valsartan are not eliminated in hemodialysis. The patients who received doses of SV 50 to 100 mg maintained the concentration ranges [94].

There is evidence on the safety and efficacy of sacubitril/valsartan in patients with CKD without evidence of hypotension or hyperkalemia. HF is a common disease in patients with CKD in renal replacement therapy, such as peritoneal dialysis, associated with a high risk of mortality and adverse cardiovascular events in the evolution of these patients compared to those who do not have heart failure [95].

Nguyen et al. conducted a meta-analysis to assess the efficacy and safety of ARNI in CKD stage 5, and the primary outcome was the change in left ventricular ejection fraction (LVEF) between baseline and post-ARNI treatment, where secondary outcomes included hospitalization for HF, mortality, and residual renal function; finding significant differences in left ventricular ejection fraction (LVEF) before and after the initiation of ARNI treatment. Patients treated with ARNI had a lower risk of all-cause mortality (relative risk [RR] 0.64; 95% CI: 0.45–0.92), and the same rate of hospitalization was observed between the groups. ARNI treatment improved left ventricular end-systolic diameter, left ventricular mass index, left atrial diameter, and the E/e′ ratio (*p* < 0.05), without significantly increasing the risk of severe hyperkalemia or symptomatic hypotension [96].

### 6.3. ACE and ARB Inhibitors

Enalapril and candesartan have been shown to reduce cardiovascular mortality and hospitalization rates in patients with heart failure with reduced ejection fraction (HFrEF) [97,98]. Telmisartan use in patients with chronic kidney disease (CKD) on hemodialysis and HFrEF demonstrated mortality and hospitalization reductions over three years compared to placebo; however, hypotension and hyperkalemia led to treatment discontinuation in some cases [99]. The efficacy of ACE inhibitors in patients with HF and CKD remains controversial, with some studies supporting their benefit and others indicating no advantage [100,101,102]. Hyperkalemia is a significant adverse effect, as evidenced by Chang et al., who reported a 30% incidence of hyperkalemia in patients with CKD stages 4 and 5, resulting in discontinuation of ACE inhibitors or ARBs in 24% of cases [103]. Despite this, the cardiovascular benefits warrant the implementation of hydroelectrolyte management strategies [104]. To maximize safety and maintain therapeutic benefits, guidelines recommend close monitoring of serum potassium and renal function at baseline, within 1–2 weeks after initiation or dose escalation, and subsequently every 1–3 months based on kidney function and risk factors. More frequent monitoring may be necessary for high-risk patients. Dose adjustments, dietary potassium restriction, potassium-lowering agents, and individualized risk–benefit assessments facilitate the safe continuation of these therapies when appropriate.

### 6.4. Steroidal and Non-Steroidal Mineralocorticoid Receptor Antagonists

The prescription rate of mineralocorticoid receptor antagonists is 33% to 45% of patients with HFrEF [105]. The prescription is frequently limited by hyperkalemia. There are studies in patients with CKD with HFrEF, both in dialysis and hemodialysis, in which hyperkalemia was observed [106,107]. There is a meta-analysis of 15 studies of patients on hemodialysis with HF who required spironolactone, observing that it reduces all causes of mortality. Although spironolactone was found to be associated with high potassium levels, it was not considered a high risk for hyperkalemia [108]. Regarding the safety of spironolactone in renal replacement therapy modality hemodialysis, the SPin-D clinical trial compared to placebo in which electrocardiographic monitoring was performed at baseline and follow-up, identifying an arrhythmia in 43% at baseline and 81% at the end of follow-up, observing a higher frequency of bradycardia and atrioventricular blocks events in the spironolactone group (82.4 versus 38.7 events/100 patient-days; *p* = 0.001) [109].

Non-steroidal mineralocorticoid receptor antagonists include finerenone, a non-steroidal molecule with high selectivity for mineralocorticoid receptors [110]. Finerenone antagonistically inhibits transcriptional cofactors involved in pro-inflammatory and pro-fibrotic pathways, distinguishing it from spironolactone and eplerenone [111]. In patients with congestive heart failure and chronic kidney disease, finerenone treatment resulted in an over 30% reduction in proBNP levels during follow-up, accompanied by decreased overall mortality and hospitalization rates compared to eplerenone [112].

Regarding the use of finerenone, the FIDELIO CKD study which included patients with chronic kidney disease in pre-dialysis without an initial or co-existing diagnosis of heart failure, through a randomized clinical trial, evaluated major adverse cardiovascular events (MACE) as well as a worsening of kidney function and the need for renal replacement therapy *(RRT).* In the group that received finerenone, hyperkalemia required discontinuing management in 110 patients, and the study showed a reduction of MACE of 14% and a deterioration of renal function of 23%, as well as a rate of congestive heart failure of 22% [113].

In the FINE ARTS study conducted by Salomon et al., with the performance of a controlled clinical trial in patients with congestive heart failure with an ejection fraction of 40% or more, to whom finerenone versus placebo was administered with a follow-up for 32 months, evaluating the primary outcome of worsening of functional class evaluated by the Kansas questionnaire, death from cardiovascular origin, and the evaluation of renal function evaluated with a sustained deterioration of more than 50% of the glomerular filtration rate or the need for inclusion in renal replacement therapy. In the finerenone group, 1083 primary outcomes occurred in 624 patients out of 3003, versus 1283 primary outcomes in 719 patients in the placebo group (HR 0.74–0.95; *p* = 0.007). The total number of worsening of the functional class of heart failure in the finerenone group was 842 versus 1024 in the placebo group (OR, 0.82; 95% CI, 0.71 to 0.94; *p* = 0.006) [114].

Regarding renal outcomes, finerenone did not reduce the risk of hyperkalemia, which was more frequent in the finerenone group; however, hospitalization rates for hyperkalemia were lower in the finerenone group (0.5% versus 0.2% in the placebo group) [115]. The mechanism of action of sodium-glucose cotransporter-2 (SGLT2) inhibitors involves the reabsorption of filtered glucose in the proximal tubule, preventing its loss from the body [116].

The trial (DAPA-CKD) has reported fewer hospitalizations for HF in patients with CKD (eGFR > 25 but <75 mL/min per 1.73 m^2^) and a reduction in proteinuria [117].

In a study evaluating the use of iSGLT2 in patients with DM and CKD (glomerular filtration rate of 20 mL/min), a regression model was used to assess the onset of renal replacement therapy, diabetic ketoacidosis, acute myocardial infarction, and mortality. showing that ISGLT2 users had a lower risk of needing dialysis (OR 0.34 [95% CI, 0.27 to 0.43]), with a reduction in hospitalization for heart failure (OR 0.80 [95% CI, 0.73 to 0.86]) [118].

There is evidence in favor of the reduction of major cardiovascular events (MACE) in the CREDENCE trial which evaluated the benefits of using canagliflozin including patients with CKD with a glomerular filtration rate of 30–90 mL/min with albuminuria greater than 300 mg, observing a reduction in the baseline glomerular filtration rate of 2.74 mL/min and observing a protective effect against MACE HR 0.80 (95% CI 0.67–0.85), as well as death or hospitalization for congestive heart failure HR (95% CI 0.57–0.87) [119].

In the EMPA-KIDNEY study, empagliflozin was evaluated, observing a reduction in the glomerular filtration rate of 1.37 mL/min, with a beneficial effect against cardiovascular death and hospitalization for heart failure [120].

Although the use of sodium-glucose cotransporter-2 (SGLT2) inhibitors in chronic kidney disease (CKD) is generally limited to patients with a glomerular filtration rate (GFR) of 25–30 mL/min, benefits have been observed in patients with GFR below 15 mL/min, particularly those at high cardiovascular risk [121]. Yen et al. evaluated SGLT2 inhibitor use in pre-dialysis patients with GFR less than 20 mL/min, assessing risks of initiating renal replacement therapy, hospitalization for heart failure, acute myocardial infarction, and euglycemic diabetic ketoacidosis via proportional hazards analysis. The study found that SGLT2 inhibitor use was associated with a reduced risk of dialysis initiation and hospitalization for heart failure, although no significant difference in mortality was observed [122].

### 6.5. Glucagon-like Peptide-1 (GLP-1) Agonist and Cardiovascular Disease

Glucagon-like peptide-1 (GLP-1) is a molecule characterized by glutamine substitution with lysine at position 26 and arginine substitution with lysine at position 34 [123]. GLP-1 receptor agonists, such as liraglutide and semaglutide, have transformed the management of diabetes and obesity. These agents have demonstrated reductions in major adverse cardiovascular events (MACE), cardiovascular mortality, and progression of nephropathy in trials such as LEADER, without significant effects on other cardiovascular outcomes [124]. The SUSTAIN 6 trial evaluated the cardiovascular safety of subcutaneous semaglutide in patients with type 2 diabetes and high cardiovascular risk, reporting a 26% reduction in MACE, 39% reduction in mortality, and 36% reduction in nephropathy progression, with no differences in other cardiovascular outcomes [125]. The PIONEER 6 study assessed oral semaglutide in patients with type 2 diabetes, high cardiovascular risk, cardiovascular disease, and chronic kidney disease (CKD), finding no reduction in MACE but significant decreases in cardiovascular death by 51% and all-cause mortality by 49% [126].

In the STEP-HFpEF trial, semaglutide was evaluated and showed a significant decrease in body weight (13.3% loss vs. 2.6% in the placebo group), and it improved the Kansas City Cardiomyopathy Questionnaire clinical summary score that assesses quality of life and 6 min walk distance, the use of GLP-1 analogues for the management of patients with obesity and HF patients [127].

Orandi et al. evaluated the safety and efficacy of GLP-1 agonists in patients with CKD and diabetes on dialysis, observing a 23% reduction in mortality among those receiving GLP-1 agonists [128]. We summarize an integrative in Table 5.

### 6.6. Other Medications

Hydralazine and isosorbide dinitrate are frequently used in patients with CKD, and they reduce afterload and blood pressure. The A-HeFT study demonstrated the benefit of hydralazine with isosorbide dinitrate in African American patients with HFrEF (10.2% mortality in the placebo group vs. 6.2% in the treatment group; *p* < 0.02). Hydralazine-Isosorbide is also associated with a decrease in the rate of first hospitalization for heart failure and an improvement in quality of life [141].

Before the era of beta-blockers, digoxin reduced hospitalizations in patients with HFrEF; however, no mortality benefit was observed. Digoxin and is eliminated primarily by the kidney, so care must be taken because it can cause a variety of brady or tachyarrhythmias [133]. In older adults with CKD starting digoxin at >0.125 versus ≤0.125 mg/d, it was associated with a higher 90-day risk of a hospital admission or an ED visit with toxicity as follows: 149 versus 33 events per 1000 person-years (wHR, 5.75 [95% CI, 4.00–8.27]). Therefore, close monitoring and follow-up allow us to assess the risks and side effects in this vulnerable population [142].

Ivabradine can be considered in patients with sinus rhythm and HFrEF who tolerate the maximum beta-blocker dose and continue with a heart rate of 70 beats per minute or greater. This recommendation is based on the SHIFT study, which found that ivabradine was associated with a decrease in hospitalizations. However, this study excluded patients with CKD [143].

## 7. Strategies for Monitoring Fluid Overload in Patients with Chronic Kidney Disease on Renal Replacement Therapy

Estimating hydration status is critically important in patients with chronic kidney disease (CKD) and heart failure, as excess volume is associated with impaired organ function and increased mortality [144].

In this context, a systematic distinction between hemodynamic and interstitial congestion has been proposed. Hemodynamic congestion is characterized by increased filling pressures and central venous pressure, with retrograde transmission of pressure to solid organs such as the liver, kidneys, and intestines. Interstitial congestion reflects the accumulation of fluid in the interstitial space and serous cavities, with clinical manifestations including peripheral edema, ascites, and pleural effusions. This decoupling between intravascular and interstitial volume explains why some patients may present with significant congestion despite not showing significant increases in interdialytic weight or evident alterations in blood pressure, leading to the concept of “subclinical congestion,” which incorporates ultrasound findings into the assessment of volume status [145,146]. Volume overload has traditionally been studied for its impact on the cardiovascular system, as it induces inflammatory changes and is considered a trigger for left ventricular hypertrophy and diastolic dysfunction. It is also associated with interstitial pulmonary edema, pleural effusion, and pulmonary hypertension.

Clinical criteria for fluid overload, such as peripheral oedema, lung auscultation, dyspnea, hypertension, and jugular vein distention, prompt us to consider adjusting dry weight in the hemodialysis population [147,148].

Currently, we have more medical devices that allow us to monitor the patient’s fluid status, such as the use of ultrasound in hemodialysis units and the use of bioimpedance, in addition to other useful biomarkers in monitoring hydro-saline congestion, since hydro-saline overload leads to an increase in hospitalization and mortality rates in the population on renal replacement therapy.

### 7.1. Bioimpedance

Bioimpedance is a non-invasive measurement of the resistance and reactance of body tissues, quantified by the application of an electric current through electrodes adhered to the skin, from which the volumes of fluid compartments and body composition are estimated [149]. Bioimpedance methods include both bioimpedance analysis (BIA) (single-frequency, multi-frequency, and bioimpedance vector analysis [BIVA]) and bioimpedance spectroscopy (BIS), both of which have been shown to be highly reproducible and have been validated with reference techniques [150].

Tabinor et al. conducted a meta-analysis to determine if fluid overload is a predictor of mortality in patients with CKD, including 60,790 patients, of which 8187 deaths occurred, with the most evaluated modality being hemodialysis and only 5% peritoneal dialysis. In relation to the bioimpedance findings, these included the phase angle/bioimpedance vector (41%), the fluid overload index (39%), and the extracellular/intracellular water ratio (20%). Fluid overload was defined by bioimpedance, which independently predicted mortality, and it demonstrated that fluid overload >15% (HR 2.28; 95% CI: 1.56–3.34; *p* < 0.001) and a decrease of 1 degree in the phase angle (HR 1.74; 95% CI: 1.37–2.21; *p* < 0.001) predicted mortality [151].

Wang and Gu published a 2021 meta-analysis whose research question was to assess the risk of fluid overload in cardiovascular events and mortality in hemodialysis and peritoneal dialysis, with the following distribution: hemodialysis in thirty-one of fifty-five studies and peritoneal dialysis in sixteen studies; four studies used a combination of the two treatments. Integrating 104,758 patients in the meta-analysis. A value of extracellular water/total body water (ECW/TBW) >0.4 (HR 5.912; 95% CI: 2.016–17.342), a value of ECW/intracellular water (ICW) for each 0.01 increase (HR 1.041; 95% CI: 1.031–1.051), and a value of overload/ECW >15% (HR 2.722; 95% CI: 2.005–3.439) increased the risk of mortality in dialysis patients. An ECW/TBW > 0.4 (HR 2.679, 95% CI: 1.345–5.339) and an ECW/ICW for 10% increase (HR 1.032, 95% CI: 1.017–1.047) were associated with a higher risk of cardiovascular events in dialysis patients [152].In congestive heart failure, different observational studies have been carried out, such as the one conducted by Rodriguez-Lopez et al., which included 100 patients with congestive heart failure to evaluate if fluid overload by bioimpedance is associated with worsening congestive heart failure in stable outpatient patients with HF and reduced LVEF, in multivariate binary logistic regression analysis, showed that fluid overload was the only independent predictor as a deterioration of the functional class and decompensations (adjusted OR: 2.7; 95% CI: 1.30–5.63; *p* = 0.008) [153].

### 7.2. Ultrasound

Point-of-care ultrasound (POCUS) is emerging as a valuable bedside tool for assessing venous congestion, with the Venous Excess Ultrasound (VExUS) technique gaining prominence. VExUS facilitates non-invasive quantification of venous congestion, relying on measurements of the inferior vena cava (IVC) size and Doppler assessments of the hepatic vein (HV), portal vein (PV), and intrarenal vein, thereby providing real-time insights into hemodynamic status and guiding therapeutic interventions [154].

The VExUS grading system categorizes congestion based on IVC diameter and Doppler findings in HV, PV, and IRV. An IVC diameter ≤2 cm indicates grade 0 (no congestion). Grades 1–3 are defined by abnormalities in HV, PV, and IRV Doppler [155].

Pulmonary ultrasound is used to evaluate volume overload by measuring extravascular lung water accumulated in the pulmonary interstitium, which produces B-lines, reverberation artifacts when ultrasonic waves reach the air-fluid interface [156]. The number of B-lines was proportionally related to the mitral gradient, left atrial volume, and maximum diameter of the inferior vena cava, and it was inversely related to the left ventricular ejection fraction (LVEF) [157].

Pulmonary ultrasound has limitations, one of which is that it is operator-dependent, so the use of technology in the interpretation of ultrasound patterns with the advent of the use of artificial intelligence (AI) in assessing clinical criteria and the use of AI as part of the tools useful for teaching and with clinical applicability [158].

Constant monitoring of the patient’s dry weight during hemodialysis is important to prevent mortality attributable to overflow [159].

In the LUST study, all patients were evaluated before and after dialysis. A thorough clinical evaluation of volume status was performed immediately before the ultrasound. B-lines, peripheral edema, dyspnea (present or absent), crackles on lung auscultation, interdialytic body weight gain, and body weight were all assessed. A meticulous evaluation was conducted to assess for crackles and peripheral edema and to rule out inflammatory or infectious bronchopulmonary disease. Simultaneously, pulmonary congestion was assessed by lung ultrasound according to the number of B-lines. Two classic physical signs, like lung crackles and peripheral edema, have a very low sensitivity for detecting interstitial lung edema in patients with CKD [160].

Lung ultrasound was repeated at least once a week until the treatment goal of fewer than 15 B-lines was achieved. Afterward, an ultrasound was performed once a month. Depending on the severity of lung congestion, nephrologists were assigned specific weight-reduction targets. All patients were followed for 12 months after randomization. The outcomes were all-cause and cardiovascular hospitalization. In the ultrasound-guided group (62 patients, 34%) and the control group (71 patients, 39%), the risk of hospitalization was similar. A post hoc analysis of repeated episodes of decompensated heart failure and repeated cardiovascular events showed a significant reduction in the incidence rate in the ultrasound-guided group [161].

## 8. Future Perspectives

The coexistence of chronic kidney disease and heart failure—both characterized by reciprocal organ dysfunction and persistent systemic inflammation—creates a formidable challenge for volume management, particularly in patients requiring renal replacement therapies such as peritoneal dialysis or hemodialysis. In this complex clinical landscape, lung ultrasound has emerged as a pivotal tool for the precise surveillance of pulmonary congestion in patients without residual urine. Beyond fluid monitoring, the integration of advanced diagnostics—ranging from microRNA-based clinical phenotyping to global strain imaging for predicting ejection fraction decline—is revolutionizing our ability to manage heart failure with greater nuance. Furthermore, therapeutic innovations such as GLP-1 receptor agonists have demonstrated significant utility in slowing disease progression for patients with concomitant kidney disease.

However, a critical evidence gap remains. While the ongoing VICTOR trial (A Study of Vericiguat in Participants with Chronic Heart Failure with Reduced Ejection Fraction) is currently evaluating the efficacy of vericiguat in stable HFrEF patients with a glomerular filtration rate above 15 mL/min, its safety and viability within the RRT population have yet to be determined.

Ultimately, the successful management of this high-risk population requires a shift toward multi-modal care. By bridging emerging molecular biomarkers, advanced imaging, and novel pharmacotherapies, clinicians can move beyond reactive volume control toward a proactive, personalized strategy that addresses the complex interplay of the cardiorenal axis.

## 9. Conclusions

This review provides clinicians with comprehensive information on managing patients with chronic kidney disease and heart failure, including the use of biomarkers and evidence-based pharmacological strategies. It underscores the necessity for further research involving this population to reduce hospitalization and mortality related to cardiovascular events. Additionally, the review emphasizes the importance of evaluating biomarkers across diverse populations to standardize reference values.

## Figures and Tables

**Table 1:** Functional classification of heart failure in hemodialysis patients.

Classification	Characteristics
**Class 1**	Patients with echocardiographic evidence of asymptomatic heart disease
**Class 2R**	Dyspnea on exercise that reverts with renal replacement therapy/ultrafiltration
**Class 2NR**	Dyspnea on exercise that does not revert with renal replacement therapy/ultrafiltration
**Class 3R**	Dyspnea with activities of daily living that reverts with renal replacement therapy/ultrafiltration
**Class 3NR**	Dyspnea with activities of daily living that does not revert with renal replacement therapy/ultrafiltration
**Class 4R**	Dyspnea at rest that reverts with renal replacement therapy/ultrafiltration
**Class 4NR**	Dyspnea at rest that does not revert with renal replacement therapy/ultrafiltration

**Table 2:** Framingham Diagnostic Criteria for Heart Failure.

Major Criteria of HF	Minor Criteria of HF
Acute pulmonary edema Cardiomegaly Hepatoyugular réflex Jugular vein distention Paroxysmal nocturnal dyspnea Pulmonary rales Third heart sound (S3 gallop)	Ankle edema Dyspnea on exertion Nocturnal cough Hepatomegaly Pleural effusion Tachycardia

**Table 3:** BNP and proBNP levels in a population with chronic kidney disease [43].

CKD Stage	BNP	Pro BNP
1	15.9 (8.3–30.7)	44.5 (23.2–98.9)
2	15.9 (8.3–30.7)	44.5 (23.2–98.9)
3	37.5 (15–83.1)	165.8 (71.7–463.5)
4	59.4 (33.2–147.4)	445.4 (216.7–939)
5	133.9 (58.2–338.6)	1686.5 (571.4–5308)

**Table 4:** Considerations regarding the interpretation of biomarkers in HF.

Biomarker	Conditions That Increase BNP Values	Conditions That Decrease and Underestimate BNP Values	Reference
**BNP**	Elderly Chronic inflammation Atrial fibrillation Use ARNI	Obesity Pericardial effusion Pericardial thickening	[60]
	**False positive**	**False negative**	
**Troponins**	Myopathies High levels of rheumatoid factor Fibrin interference Elevated alkaline phosphatase	Hyperbilirubinemia Lipemia Biotin Hemolysis	[61]
**Galectin 3**	**Conditions that increase galectin 3 values**	**Relationship with kidney disease**	
	Chronic inflammatory conditions	Lupus nephritis IgA nephropathy Polycystic kidney disease	[62,63,64]
**ST2**	**Conditions that increase ST2 values**	**Findings in murine models with relevance to CKD and AKI**	
	Infections Chronic inflammatory conditions	Inflammation Fibrosis	[62,65,66]
**GDF 15**	**Conditions that increase BNP values**		
	Elderly Cancer Metabolic diseases	Poor prognosis across several solid tumors, including colorectal, gastric, pancreatic, breast, lung, prostate, and head and neck cancers	[62,67]
	**Elevation under benign conditions**	**Elevation in malignant conditions**	
**CA 125**	Endometriosis Benign ovarian tumors Salpingitis Pelvic inflammatory disease	Ovarian cancer Endometrial cancer Lung cancer Breast cancer Lymphoma	[68]
	**Chronic disease**		
	Liver cirrhosis Hepatitis Pancreatitis Tuberculosis Pericardial disease		

**Table 5:** Integrative management of heart failure in CKD patients.

CKD Stage	ACE and ARB Inhibitors	Beta-Blockers	Angiotensin Receptor-Neprilysin Inhibitors (ARNI)	SGLT2 Inhibitors	Steroidal Mineralocorticoid Receptor Antagonists	Non-Steroidal Mineralocorticoid Receptor Antagonists	Glucagon-like Peptide-1 (GLP1) Agonist	Diuretics
1	✓	✓	✓	✓	✓	✓	✓ ∞	✓
2	✓	✓	✓	✓	✓	✓	✓ ∞	✓
3	✓	✓	✓	✓	✓	✓	✓ ∞	✓
4	✓ ±	✓	✓ *β	✓	✓	✓±	✓ ∞	✓
5 conservative management	✓ ±	✓	✓ *β	✓ #	X	✓±	✓ ∞	✓ #
Hemodialysis	✓ ±	✓ *	✓ *β	✓ #	X	≠	✓ ∞ +	✓ #
Peritoneal	✓ ±	✓	✓ *β	✓ #	X	≠	✓ ∞ +	✓ #
Effect on kidney function	Early decline in GFR after initiation approximate 6.4 mL/min/1.73 m^2^) [129,130]	No decline GFR [131]	Early decline in GFR after initiation approximate (0.5–1 mL/min/1.73 m^2^) 123, A 31% reduction in the odds of renal impairment with ARNI compared with ARB [132,133]	Early decline in GFR after initiation approximate (0.3–4 mL/min/1.73 m^2^) [134,135]	Early decline in GFR after initiation approximate (2.3–6.7 mL/min/1.73 m^2^) [136]	Stop decline in GFR after initiation [137]	No decline GFR [138,139]	No decline GFR, GFR increased in subjects with reduced GFR at baseline [140]
Reduction cardiovascular mortality	Yes	Yes	Yes	Yes 126	Yes in CKD stage 3 In 5 CKD stage increases risk of bradycardia and atrioventricular blocks events	Yes	Yes	No

± potassium monitoring, * monitoring of intradialytic hypotension, # consider whether the patient retains residual diuresis, ≠ lack of inclusion of patients with stage 5 CKD, ∞ monitoring of gastrointestinal tolerance, assessing hydration status, + benefit in patients with dialysis and diabetes, *β* needs dose titration, ✓ use, X Its use is not recommended.

## Data Availability

No new data were created or analyzed in this study. Data sharing is not applicable to this article.
